# On the Aging Kinetics of a Flame-Resistant AZ91D-1.5%Ca Magnesium Alloy Processed with Ultrasonic Vibration

**DOI:** 10.3390/ma16083152

**Published:** 2023-04-17

**Authors:** Inês V. Gomes, Fabrizio D’Errico, José L. Alves, Hélder Puga

**Affiliations:** 1 CMEMS—UMinho, Department of Mechanical Engineering, Campus of Azurém, University of Minho, 4800-058 Guimarães, Portugalpuga@dem.uminho.pt (H.P.); 2 LABBELS—Associate Laboratory, 4800-058 Guimarães, Portugal; 3 Department of Mechanical Engineering, Politecnico di Milano, Via La Masa 34, 20156 Milan, Italy

**Keywords:** magnesium alloys, ultrasound treatment, solution, aging, Mg_17_Al_12_

## Abstract

The Mg-Al-Zn-Ca system has demonstrated excellent flame resistance and mechanical properties in the as-cast condition. However, the potential of these alloys to be heat-treated, e.g., by aging, as well as the influence of the initial microstructure on the precipitation kinetics, is yet to be comprehensively explored. Ultrasound treatment was applied during the solidification of an AZ91D-1.5%Ca alloy to promote microstructure refinement. Samples from treated and non-treated ingots were subjected to solution treatment at 415 °C for 480 min, followed by aging at 175 °C for up to 4920 min. The results showed that the ultrasound-treated material could reach the peak-age condition in a shorter period than the non-treated one, suggesting accelerated precipitation kinetics and, thus, enhanced aging response. However, the tensile properties showed a decrease in the peak age compared to the as-cast condition, probably due to the formation of precipitates at the grain boundaries that promote the formation of microcracks and intergranular early fracture. This research shows that tailoring the material’s as-cast microstructure may positively affect its aging response, shortening the heat treatment duration, thereby making the process less expensive and more sustainable.

## 1. Introduction

The trade-off between magnesium alloys’ strength and ductility is still a challenge that has been addressed by several authors in the last few years [1,2]. Significant attention has been paid to wrought magnesium alloys, resulting in substantial advances [3,4,5], while casting ones have been neglected concerning their processing and optimization [6]. 

A considerable fraction of intermetallic phases form during the solidification process of the alloys, which can play a role in the material’s behavior. The intermetallics’ morphology and distribution can thus strongly influence the material’s mechanical and corrosion properties, possibly optimizing its performance by tailoring it. As the predominant intermetallic phase in AZ91D magnesium alloy, *β*-Mg_17_Al_12_ precipitates may enhance the material’s strength, but at the expense of its ductility. The precipitation of this phase from the supersaturated matrix may assume two different and competitive modes: discontinuous and continuous precipitation. Discontinuous precipitates exhibit an elliptical or lamellar shape composed of *β*-Mg_17_Al_12_ and aluminum-enriched *α*-Mg and form mainly at the grain boundaries, growing inward [7]. Continuous precipitates, on the other hand, are characterized by a lozenge-shaped plate phase known as the Widmanstätten phase, which nucleates and grows inside the α-Mg grains with a primary habit plane parallel to the [0001] basal plane of the matrix [8]. The contribution of each precipitate type to the material’s mechanical properties is not yet fully understood, and the literature offers conflicting views. Some authors [9,10] claimed that continuous precipitates could endow the material with a superior age-hardening response, given that discontinuous precipitate morphology could lead to early fracture. From a different perspective, discontinuous precipitates were suggested to play the leading role in aging hardening due to the continuous precipitates presenting thin plates parallel to the basal plane, easing the dislocation gliding [7,11].

Solution treatment followed by artificial aging has proven to be able to tailor the morphology of the *β*-Mg_17_Al_12_ precipitates by controlling both the temperature and duration of the heat treatment [12,13,14]. However, it has been noted that magnesium alloys aged at temperatures below 200 °C require long aging periods to attain their peak-aged condition [15], making the process energy-intensive and expensive. On the other hand, ultrasound treatment has been intensively explored for microstructural modification, and it has been demonstrated that it can promote peak-aging conditions in a shorter period in aluminum alloys [16]. Moreover, ultrasonic excitation may grant a refined microstructure with a smaller grain size [17] as well as the formation of vacancies [18], features deeply related to the formation of discontinuous and continuous precipitates, respectively [9]. The potential of ultrasonic vibration to assist in several manufacturing techniques is known, namely in additive and hybrid manufacturing areas. It has been shown that ultrasonic vibration promotes higher powder utilization efficiency, lower surface roughness and microstructural improvement [19], the reason why it can be a path to boost the application of additive and hybrid manufacturing in high-quality standards industries [20].

Although some research has already been published on the aging kinetics of different magnesium alloys, there is still a lack of information regarding the Mg-Al-Zn-Ca system. These alloys have demonstrated excellent results of ignition resistance allied to excellent strength-to-weight ratio, granting an increasing interest in them recently [21,22]. However, adding calcium is known to lead to the formation of thermally stable Al-Ca intermetallics, changing the fraction of the *β*-Mg_17_Al_12_ phase formed during solidification and heat treatment [23]. 

This work details the effect of the as-cast microstructure of an AZ91D-1.5%Ca (wt.%) on its age-hardening response. In this sense, ultrasound treatment was applied to the material during its solidification, refining its microstructure. The heat-treated samples’ hardness evolution was investigated for non- and ultrasound-treated (US-treated) conditions, and the precipitates formed were comprehensively characterized. In addition, the tensile mechanical properties of the material in the as-cast, solution-treated and peak-aging states were determined at room temperature. 

We hypothesize that ultrasound treatment can accelerate the precipitation kinetics, helping to reduce the heat treatment duration and temperature and thus contributing to a more economical and environmentally sustainable manufacturing chain.

## 2. Materials and Methods

A SiAlON crucible was used to melt an AZ91D-1.5%Ca alloy (Table 1) in a resistance furnace under an Ar protective atmosphere. 

A previous stage at 450 °C was carried out to prevent melt contamination and eliminate humidity and moisture from the crucible and tools used. The melt was heated to 620 ± 5 °C and held for 20 min to form a CaO protective layer, after which the temperature was increased to 660 ± 5 °C and held for 10 min for homogenization. The melt was then poured into a pre-heated to 350 ± 5 °C metallic mold coupled to an ultrasound device that transmitted acoustic energy to the medium until it reached 525 ± 5 °C. Experiments were also conducted without the use of ultrasonic vibration as a comparison. The samples were subjected to a 480 min heat treatment at 415 °C followed by quenching in water at room temperature to avoid additional phase transition induced by residual heat. Artificial aging was then carried out at 175 °C for 240 to 4920 min.

The microstructural characterization and hardness testing samples were ground with gradually finer SiC papers and polished with a 1 μm polycrystalline diamond solution, followed by oxide polishing with 0.02 μm colloidal silica. A 4% solution of HNO_3_ in ethanol was used to etch the samples before optical microscope examination (LEICA DM2500 M). A deep etching technique based on the selective dissolution of the matrix was applied to study the intermetallic shape. The morphology and composition of the phases present in the microstructure were detailed further through scanning electron microscopy with energy dispersive spectroscopy (SEM-EDS Phenom XL2, Thermo Fisher Scientific, Massachusetts, USA). Hardness measurements were conducted in an Officine Galileo Mod using a D200 tester under a load of 50 gf. A minimum of five indentations were averaged for each reported hardness measurement. Casted samples were machined into cylindrical tensile specimens (type D, according to ISO 6892-1) with an 8.00 ± 0.08 mm diameter and a 50.00 ± 0.50 mm proof length. Universal testing equipment (INSTRON 8874) was used to perform tensile testing with a 1 mm/s displacement rate until the specimens’ fracture occurred and a load drop was observed. Three samples were tested for non- and US-treated alloy in the as-cast, solutionized, and peak-aging conditions.

## 3. Results and Discussion

**Figure 1:**
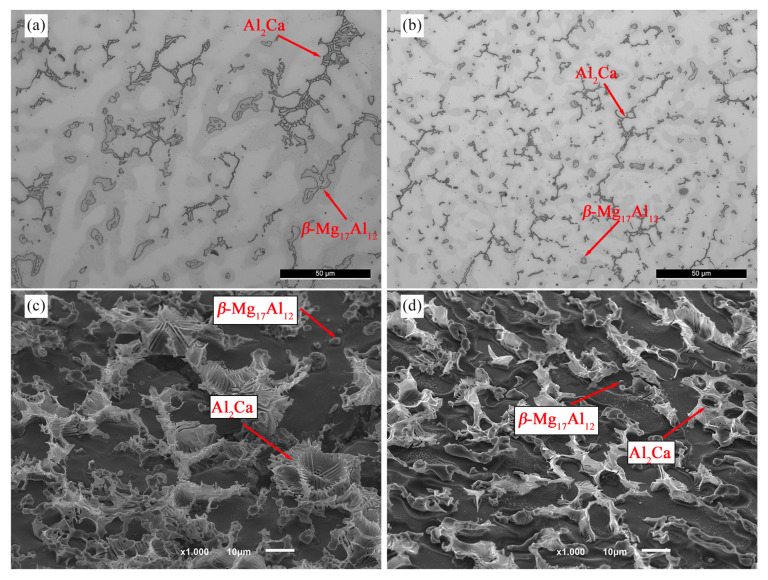
Microstructures of the (**a**,**c**) non- and (**b**,**d**) US-treated samples in the as-cast condition.

Figure 1a,b present the optical micrographs of the non- and US-treated samples in the as-cast condition.

Regardless of the processing procedure, the microstructure of the as-cast samples is composed of *α-*Mg, *β*-Mg_17_Al_12_, Al_2_Ca, and Al-Mn rich phases, as observed in our previous work [23]. However, significantly different morphologic characteristics could be observed, which may be attributable to the application of ultrasonic vibrations to the solidifying melt. It is suggested that ultrasound treatment has stimulated the refinement of the intermetallic phases, as previously described [24,25,26]. Indeed, compared to the non-treated sample (Figure 1a), *β*-Mg_17_Al_12_ appeared smaller and more rounded in the US-treated sample (Figure 1b), while Al_2_Ca was fragmented, exhibiting a script-like disconnected morphology and a more uniform distribution. 

Color differences between the dendrite interior and exterior indicate aluminum segregation during the solidification of the alloy. Hence, the Al-rich outer regions showed a lower etching rate compared to the interior areas, which has also been referred to by Esgandari et al. [27]. 

Deeply etched microstructures (Figure 1c,d) enabled a more detailed examination of the intermetallic morphology, revealing that both non- and US-treated samples exhibited a lamellar Al_2_Ca phase with distinct morphological features. While the Al_2_Ca intermetallic displayed a rosette-like structure in the non-treated sample, the US-treated sample had a platelet morphology that developed epitaxially from the *α*-Mg phase. 

**Figure 2:**
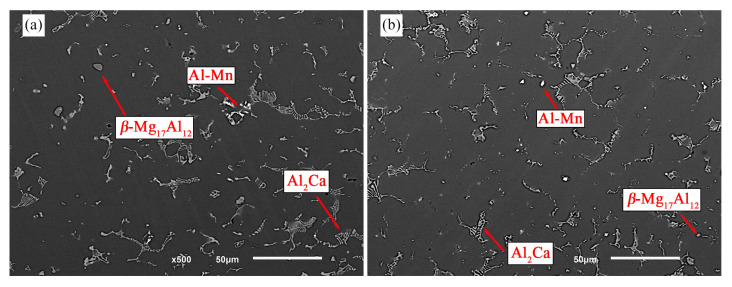
Microstructure of (**a**) non- and (**b**) US-treated samples after solution treatment at 415 °C for 480 min.

The microstructure of the solution-treated samples is presented in Figure 2. After solution treatment for 480 min at 415 °C, *β*-Mg_17_Al_12_ was nearly fully dissolved in the US-treated sample, whereas several particles could still be observed in the non-treated one. Thus, it is hypothesized that the dissolution process progressed differently depending on the processing conditions. The discrepancies observed in the materials’ response to the solution treatment may have been promoted by their different as-cast microstructures. The non-treated sample exhibited *β*-Mg_17_Al_12_ coarse bulk particles, which led to its reduced dissolution due to a slower dissolving rate, conversely to the fine intermetallic found in the US-treated sample.

Therefore, differences in the size and distribution of intermetallic particles may explain their distinct dissolution kinetics. The non-treated sample presented larger intermetallic particles and a higher aluminum concentration in their vicinity, leading to a slower dissolution rate promoted by the lower gradient. In addition, once the intermetallic volumetric fraction is comparable across the processing conditions, the intermetallics’ interfacial area of the non-treated sample is smaller and hinders the aluminum atoms’ diffusion to the matrix, slowing down the dissolution process.

On the contrary, the US-treated sample was characterized by smaller and uniformly dispersed *β*-Mg_17_Al_12_ particles, shortening the distance over which diffusion occurred and thereby the time required for the dissolution to occur.

The Al_2_Ca phase appeared not to suffer significant dissolution, possibly because of its excellent thermal stability [28,29], which limited the aluminum atoms available to migrate to the *α*-grains.

**Figure 3:**
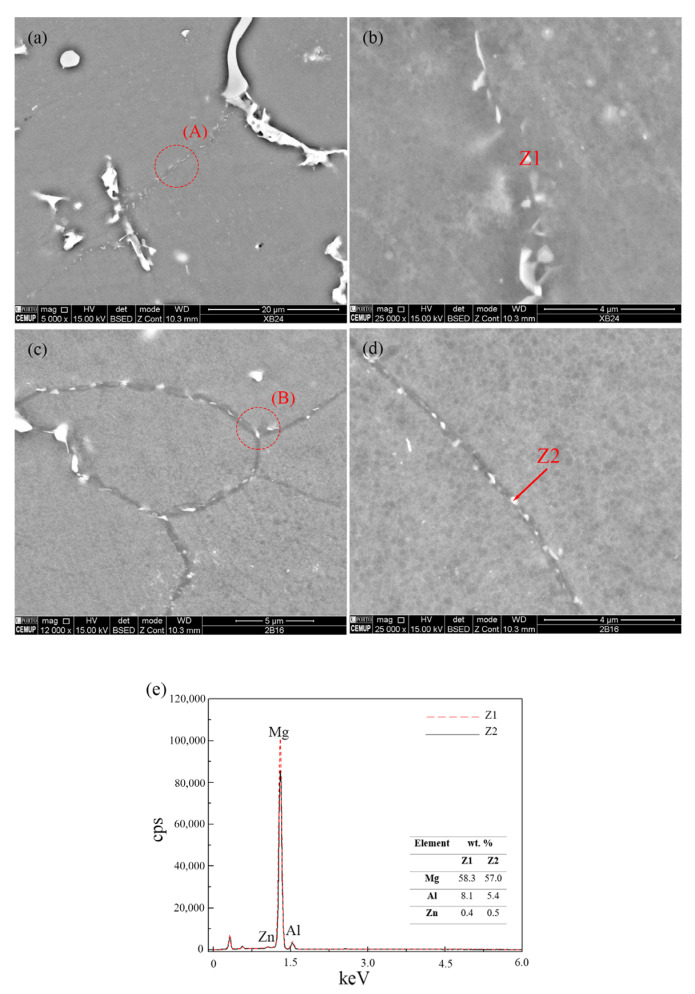
SEM micrographs of the discontinuous *β*-Mg_17_Al_12_ precipitates found in (**a**,**b**) non- and (**c**,**d**) US-treated samples, after aging at 175 °C for 1440 and 960 min, respectively; (**b**,**d**) higher magnification images of areas A and B, respectively, (**e**) EDS analysis of the particles identified as Z1 and Z2 in (**b**,**d**).

SEM micrographs of the non- and US-treated samples at the peak-age condition at 175 °C (1440 and 960 min, respectively) are shown in Figure 3, along with the EDS analysis results of the identified precipitates.

**Figure 4:**
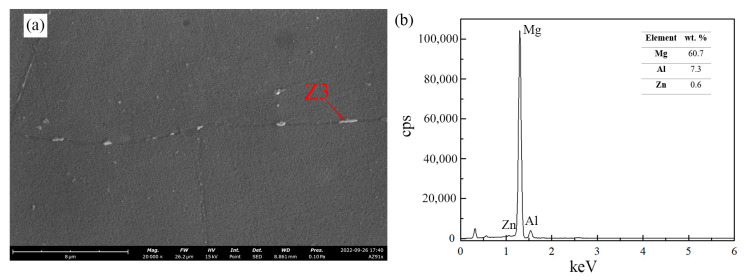
SEM micrograph of *β*-Mg_17_Al_12_ bulk precipitates found at grain boundaries of non-treated material after aging at 175 °C for 1440 min; (**b**) EDS analysis of the Z3 particle identified in (**a**).

During the aging treatment, two types of precipitates—continuous and discontinuous—may form and develop competitively as they nucleate and grow at different rates [30,31]. Although their chemical composition is the same, their morphology differs, making their strengthening effect distinct. Both non- and US-treated samples exhibited only discontinuous precipitates, which may be explained by the low aging temperature adopted [27]. Most precipitates were found along the grain boundaries—intergranular precipitates—and presented an elliptical shape. The different processing conditions are suggested to result in the modification of the precipitates, not primarily of their shape, but of their size and number density. Indeed, the non-treated sample was characterized by a lower number of precipitates whose size was larger than that of the US-treated samples. In addition, it is possible that the precipitates grew and coalesced to form massive bulk structures, as shown in Figure 4.

The US-treated sample showed a higher number of smaller *β*-Mg_17_Al_12_ precipitates distributed along the grain boundaries, which may result from the finer microstructure, i.e., the materials’ smaller grain size. The grain refinement of the alloy results in increased grain boundaries, providing more nucleation sites for *β*-Mg_17_Al_12_ discontinuous precipitates and thereby promoting faster-aging kinetics [9,32].

**Figure 5:**
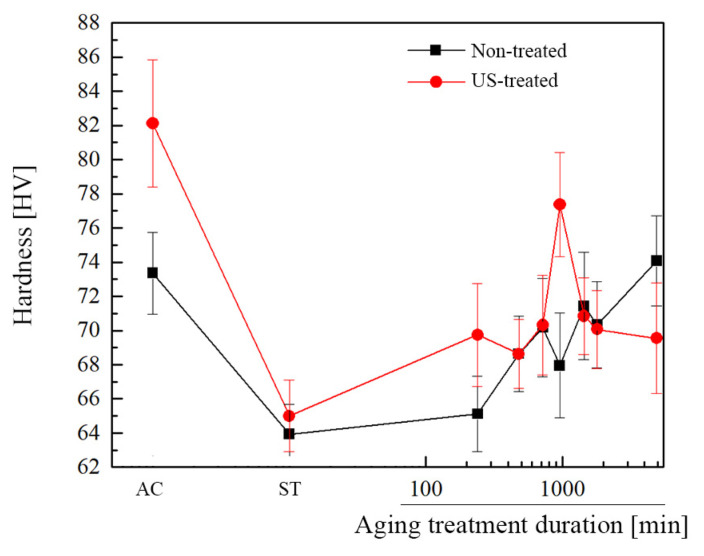
Average Vickers hardness of the non- and US-treated samples during artificial aging.

Figure 5 shows the hardness evolution under as-cast, solution-treated, and artificially aged conditions for non- and US-treated samples.

In the as-cast condition, the US-treated alloy exhibited higher hardness than the non-treated one, possibly due to its finer microstructure. Indeed, the refinement of the *α*-Mg and intermetallic phases substantially affects the material’s mechanical properties, namely its hardness. Increasing grain boundaries led to an increase in the density of small precipitates, thus improving the mechanical properties. In the non-treated samples, however, the coarser morphology of the precipitates resulted in an increased interspacing between them. Under these conditions, there is a reduced capacity to inhibit dislocation movement, so the material’s hardness is lower [33]. Similar findings were reported by Lai et al. [15], who studied the effect of aging conditions on the morphology of the *β*-Mg_17_Al_12_ precipitates and concluded that numerous smaller precipitates provided higher peak hardness. 

Due to the precipitates’ dissolution after solution treatment, which promotes material softening [34], the material’s hardness dropped dramatically, regardless of the processing conditions. The US-treated alloy hardness remained higher than that of the non-treated one in the solutionized condition due to the grain boundary strengthening effect granted by the grain refinement. At this stage, no contribution of precipitation strengthening was considered since most precipitates were dissolved after solution treatment. On the other hand, a more gradual decline in the hardness in the non-treated material may be attributable to the presence of undissolved *β*-Mg_17_Al_12_ intermetallic, whose size has decreased throughout the procedure, favoring its ability to prevent dislocation movement. 

The hardness curves’ behavior of the non- and US-treated alloys indicates that the processing conditions may profoundly influence the material’s aging response. Although the typical hardness increase was noticeable in both non- and US-treated samples, the former presented a longer incubation period, with the hardness value remaining almost constant until aging for 240 min. In contrast, the short incubation period of the US-treated alloy suggests accelerated aging kinetics of *β*-Mg_17_Al_12_ precipitates compared to the non-treated sample. Similar results were reported by Wang et al. [32]. The authors investigated whether the manufacturing process—high-pressure die casting or rheo-diecasting—influenced the material’s aging response and determined that the latter showed faster dissolution during the solution treatment and accelerated aging kinetics. 

After the incubation stage, the non- and US-treated samples’ hardness increased, although at a higher rate for the latter. The US-treated alloy reached its peak-aged condition at 960 min, following which its hardness value decreased sharply, whereas the non-treated alloy hardness increased throughout the time range considered. The larger grain size is suggested to significantly reduce the nucleation rate, delaying the material’s aging response and resulting in the non-treated sample not achieving the peak-aging condition within the time interval considered [33]. Even so, and for comparison’s sake, aging treatment for 1440 min was hereafter referred to as the peak-aging condition of the non-treated sample, corresponding to the highest hardness value observed within the heat treatment period considered. After 4920 min of aging, the non-treated samples exhibited a value comparable to that of the as-cast condition (74 HV vs. 73 HV) and still showed a tendency toward increasing hardness, which indicates that peak-aging has not been reached yet and could be higher. However, the US-treated sample showed the highest hardness in the as-cast condition (82 HV), with peak-aged hardness 5.7% lower (77 HV). According to Zhang et al. [35] and Dumpala et al. [36], the coarsening of grains during the solution treatment may be responsible for this behavior. 

Nonetheless, the ultrasound treatment of magnesium alloy has demonstrated the potential to refine the microstructure, which has proven effective in shortening the time needed for reaching the peak-aged state. In addition, the difference between the non- and US-treated samples aged for 240 min is superior to that observed in the solution-treated condition. This behavior supports the hypothesis that ultrasonic processing plays a role in precipitation behavior by anticipating it. In this sense, the increased hardness value obtained for the US-treated sample may have resulted from accelerated precipitation and grain boundary strengthening combined, consistent with Kim et al.’s findings [37]. Even so, no increase in hardness was seen after the aging treatment compared to the as-cast condition for either processing method. These results align with those of Suzuki et al. [30] and Bamberger et al. [31] for Mg-Al-Ca and Mg-Ca-Zn alloys, respectively, demonstrating the poor age-hardening response of these alloys. 

Furthermore, due to the formation of the Al_2_Ca phase during the alloy solidification, there is a low fraction of *β*-Mg_17_Al_12_ phase in the as-cast state and reduced aluminum content on the α-Mg phase, limiting the solute availability after solution heat treatment. In this sense, given the direct relationship between the transformation rate during the aging treatment and the solute atoms amount available in the supersaturated matrix, lower peak hardness values are obtained by calcium-containing magnesium alloys compared with those without this element [27].

Several studies show the ability of ultrasound treatment to enhance the mechanical properties of magnesium alloys, namely AZ91D [17]. However, whether as-cast microstructure affects the tensile behavior of heat-treated materials is still unknown. 

The results of the tensile tests performed on the non- and US-treated AZ91D-1.5%Ca (wt.%) in the as-cast, solutionized (T4), and peak-aged conditions are presented in Table 2.

The results demonstrate that ultrasound treatment significantly increased mechanical performance under all the tested conditions. In the as-cast state, the tensile strength of the US-treated material was about 50% higher than that of the non-treated one, stressing the grain refinement’s role in the material’s mechanical resistance. In addition, the yield strength was higher than in non-treated samples, but the difference was less pronounced. The increased elongation at fracture constitutes the most impressive result, enhancing by about 72.5% when ultrasound treatment is applied. The microstructure characteristics may thus be considered a critical factor for the static mechanical properties of the alloy. The conjunction of smaller grain size and refined and more uniform distributed intermetallic phases grant remarkably improved mechanical properties. Conversely, the continuous network of brittle *β*-Mg_17_Al_12_ phase detected in the non-treated sample leads to poorer mechanical properties [24]. According to Du et al. [38], such modifications decrease the stress concentration points, improving ductility by preventing early fracture. 

The solutionized samples exhibited higher elongation, yield and tensile strength than the as-cast condition, regardless of the processing route. For the non-treated sample, the increase in the yield and tensile strength was higher than that of the elongation, while the contrary was observed in the US-treated samples. The partial dissolution of the coarse precipitates may underlie this behavior, given that they constitute preferential sites for fracture initiation and act as a continuous easy crack path [39]. In addition, the dissolution of these precipitates occurred to a greater extent in the US-treated sample, leading to the matrix being dopped with aluminum atoms, easing the basal-plane deformation twinning in the magnesium alloys and thereby enhancing the elongation. 

In the aged condition, non- and US-treated materials showed a decrease in yield and tensile strength, as well as elongation. Such a loss of the materials’ mechanical properties may be promoted by forming precipitates which, contrary to what is observed for aluminum alloys, may harm the mechanical resistance. In fact, under the considered aging conditions, discontinuous precipitates formed at the grain boundaries, showing small interface bonding strength and weakening the bonding force between grains [40]. Under these conditions, the microcracks are suggested to form preferentially in the *β*-Mg_17_Al_12_/*α*-Mg, interfaces, promoting early intergranular fracture due to their growth and propagation along the grain boundaries [41]. In agreement with these findings, the higher yield and tensile strength and elongation values observed in the US-treated material after solution treatment compared to the peak-age properties appear to confirm that the precipitation of the *β*-Mg_17_Al_12_ has an overall deleterious effect on the mechanical behavior of the material. Moreover, the elongation decline showed by the non-treated material after aging may be explained by the presence of coarse intermetallic particles that did not fully dissolve and are likely to crack at low strains. This detrimental effect did not significantly compromise the ductility in the solution-treated condition due to the softer matrix, which worked to delay the cracking [42] but played a critical role in decreasing the material’s mechanical strength after aging treatment. 

According to the obtained results, it can be concluded that AZ91D-1.5%Ca (wt.%) has a limited aging potential under the considered conditions. In fact, no significant improvement in the material’s mechanical properties could be achieved through the adopted heat treatment scheme. Such a poor aging response may be explained mainly by two factors: (i) the addition of calcium promotes the formation of the Al_2_Ca intermetallic phase during the material solidification and suppresses the formation of the *β*-Mg_17_Al_12_ phase. Given its high thermal stability, the Al_2_Ca phase did not dissolve during the solution treatment, causing the aluminum content available to precipitate during subsequent aging treatment to be extremely low; on the other hand, (ii) the discontinuous precipitates formed along the grain boundaries consumed the available solute content, promoting the depletion of continuous precipitation, which could further improve the mechanical performance of the material [43]. 

Optimizing the solution treatment to dissolve the Al_2_Ca phase and applying ultrasound treatment during the casting process may constitute a route to enhance the aging response of the AZ91D-1.5%Ca (wt.%) alloy. In fact, applying ultrasonic vibration during the material solidification can promote an excess of vacancies, which act as nucleation sites for continuous precipitates [18]. Such conditions, together with a higher available aluminum content granted by the dissolution of the Al_2_Ca intermetallic, can enhance the material’s response to the aging treatment by tailoring the morphology of the resultant precipitate.

## 4. Conclusions

In this research, ultrasound treatment was used during the solidification of an AZ91D-1.5%Ca to modify its microstructure, which was then investigated for its effect on aging kinetics. The following conclusions could be drawn from the performed study:

- Applying ultrasound treatment during AZ91D-1.5%Ca (wt.%) alloy cooling has significantly changed its microstructure, promoting the refinement of *β*-Mg_17_Al_12_ and Al_2_Ca intermetallic phases.
- The refined microstructure of the US-treated sample yielded a higher hardness than that of the non-treated one in the as-cast condition.
- US-treated samples showed accelerated aging kinetics since precipitation hardening occurred for a shorter heat treatment duration compared to that of non-treated ones.
- The hardness curve of the non-treated material suggests that peak aging was not achieved under the tested conditions, which indicates that aging for periods longer than 4920 min may be required. Conversely, US-treated samples appeared to reach the peak-aging state after 960 min.
- Ultrasound treatment enhanced the ultimate tensile strength and elongation in all the considered conditions—as-cast, solutioned and aged—compared to the absence of treatment. However, the tensile properties showed a decrease in the peak age, possibly due to the formation of precipitates at the grain boundaries that promote the formation of microcracks and intergranular early fracture.

## Figures and Tables

**Table 1:** Chemical composition of AZ91D-1.5% Ca alloy (wt.%).

Alloy	Mg	Al	Zn	Mn	Ca
AZ91D-1.5%Ca	Bal.	9.7	0.5	0.2	1.5

**Table 2:** Mechanical properties of the non- and US-treated material in the as-cast, solution-treated and peak-aged conditions.

Test	Condition	Yield Strength (MPa)	Tensile Strength (MPa)	Elongation at Break (%)
Non-treated	As-cast	89 ± 5	110 ± 7	1.75 ± 0.34
	T4	137 ± 7	146 ± 8	2.04 ± 0.93
	T6–Peak-age condition (1440 min)	100 ± 4	115 ± 4	1.73 ± 0.71
US-treated	As-cast	125 ± 8	164 ± 6	3.02 ± 0.25
	T4	158 ± 6	204 ± 8	4.31 ± 1.34
	T6–Peak-age condition (960 min)	142 ± 6	169 ± 8	2.69 ± 0.89

## Data Availability

Not applicable.
